# Consolidative cystectomy after complete response to enfortumab vedotin plus pembrolizumab in metastatic bladder cancer: a case report

**DOI:** 10.1093/jscr/rjag435

**Published:** 2026-06-10

**Authors:** Romain Lefranc, Clémentine Mathieu, Achraf Ghorbel, Alexandre Léger-Vigot, Xavier Tillou, Thibaut Waeckel

**Affiliations:** Urology Department, CHU de Caen, avenue de la Côte de Nacre, 14000 Caen, France; Oncology Department, CHU de Caen, avenue de la Côte de Nacre, 14000 Caen, France; Urology Department, CHU de Caen, avenue de la Côte de Nacre, 14000 Caen, France; Oncology Department, CHU de Caen, avenue de la Côte de Nacre, 14000 Caen, France; Pathology Department, CHU de Caen, avenue de la Côte de Nacre, 14000 Caen, France; Urology Department, CHU de Caen, avenue de la Côte de Nacre, 14000 Caen, France; Urology Department, CHU de Caen, avenue de la Côte de Nacre, 14000 Caen, France

**Keywords:** bladder cancer, metastatic disease, enfortumab vedotin, pembrolizumab, consolidative surgery, pathological complete response

## Abstract

Metastatic muscle-invasive bladder cancer carries a poor prognosis despite recent therapeutic advances. Enfortumab vedotin plus pembrolizumab (EVP) is now the standard first-line treatment for metastatic urothelial carcinoma. However, the role of local consolidative treatment after deep systemic response remains undefined. We report the case of a 59-year-old man with *de novo* metastatic bladder cancer (cT3N1M1) treated with first-line EVP. After 10 cycles, imaging demonstrated a sustained complete metabolic response. Consolidative radical cystoprostatectomy with Bricker diversion confirmed a complete pathological response (ypT0N0M0R0). This case highlights that deferred local consolidative surgery may be considered in carefully selected metastatic patients achieving durable responses to modern systemic therapies.

## Introduction

Muscle-invasive bladder cancer (MIBC) with distant metastases remains associated with poor prognosis despite recent therapeutic advances. The combination of enfortumab vedotin plus pembrolizumab (EVP) has recently become the standard first-line treatment for metastatic urothelial carcinoma following the results of the EV-302 trial [1, 2]. However, the role of local consolidative surgery after deep systemic response remains unclear [3, 4]. We report the case of a patient with de novo metastatic muscle-invasive bladder (mMIBC) treated with EVP followed by consolidative radical cystoprostatectomy (RCP), which confirmed a pathological complete response (pCR).

## Case presentation

A 59-year-old man with no significant past medical history and an ECOG performance status of 1 was admitted in January 2025 for acute renal failure associated with hyperkalemia (serum creatinine >1000 μmol/l, potassium 6.2 mmol/l). Contrast-enhanced computed tomography (CT) revealed bilateral ureterohydronephrosis secondary to trigonal bladder wall thickening.

Transurethral resection of the bladder (TURB) demonstrated a large trigonal and cervical tumor involving both ureteral orifices. Histopathological examination showed invasive urothelial carcinoma, at least pT2 stage, with a 40% sarcomatoid component and associated carcinoma *in situ*. PD-L1 expression was positive with a tumor proportion score of 10%.

Initial staging with CT and ^18^F-fluorodeoxyglucose positron emission tomography/computed tomography (FDG PET/CT) demonstrated extension to the perivesical fat, a hypermetabolic right internal iliac lymph node, and a hypermetabolic manubrial bone lesion, consistent with *de novo* mMIBC (cT3N1M1) (Fig. 1).

**Figure 1:**
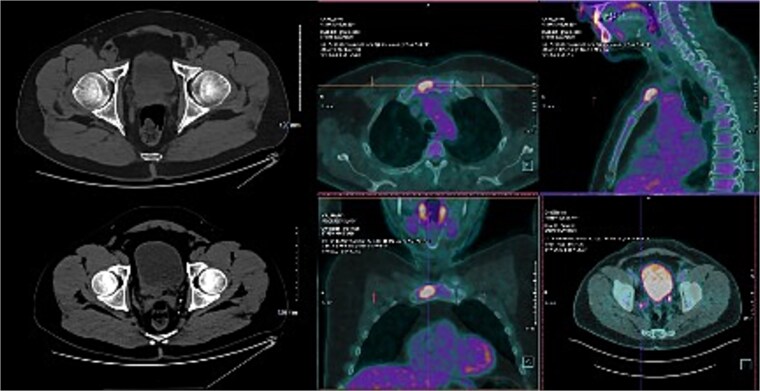
Baseline CT scan and PET/CT showing a hypermetabolic bladder tumor with pelvic lymph node involvement (right internal iliac node) and a hypermetabolic manubrial bone metastasis, consistent with *de novo* metastatic urothelial carcinoma.

Following TURB and urinary diversion, renal function improved with a creatinine clearance of 49 ml/min. After multidisciplinary tumor board discussion, first-line treatment with EVP was initiated.

After 3 months of treatment, follow-up PET/CT demonstrated a metabolic complete response (mCR) with disappearance of all previously hypermetabolic lesions. The manubrial lesion evolved toward a non-hypermetabolic osteosclerotic change. This complete response was confirmed at 6 months on subsequent imaging (Fig. 2).

**Figure 2:**
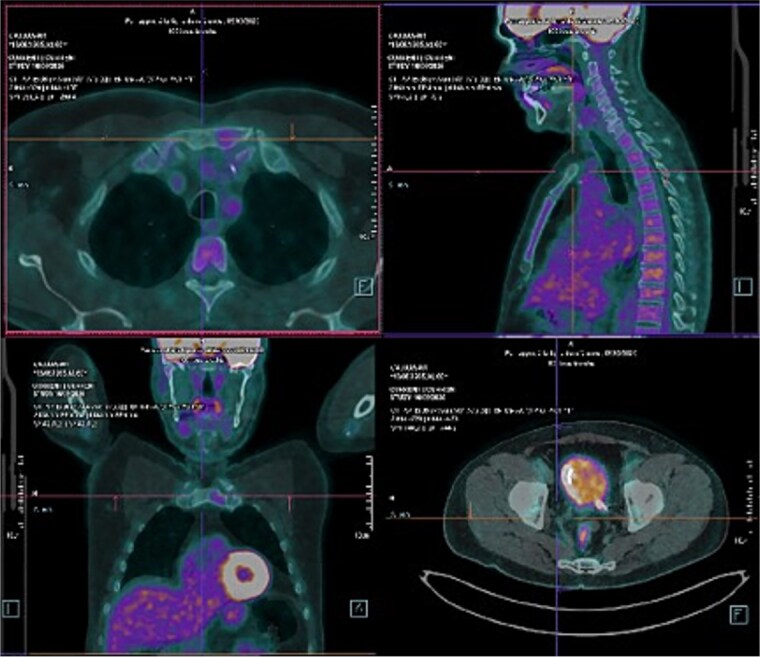
Follow-up PET/CT after 3 months of EVP demonstrating mCR, with disappearance of all previously hypermetabolic lesions. The manubrial lesion evolved toward a non-hypermetabolic osteocondensation.

During systemic treatment, the patient developed grade II nausea requiring dose reduction of enfortumab vedotin (EV) to 1.0 mg/kg, and grade II hypothyroidism requiring initiation of levothyroxine (75 μg/day). Quality of life and performance status remained unchanged.

After 10 cycles of EVP, consolidative RCP with ileal conduit urinary diversion (Bricker) was performed. Pathological examination of the surgical specimen demonstrated a pCR with no residual tumor identified (ypT0N0M0R0) (Fig. 3).

**Figure 3:**
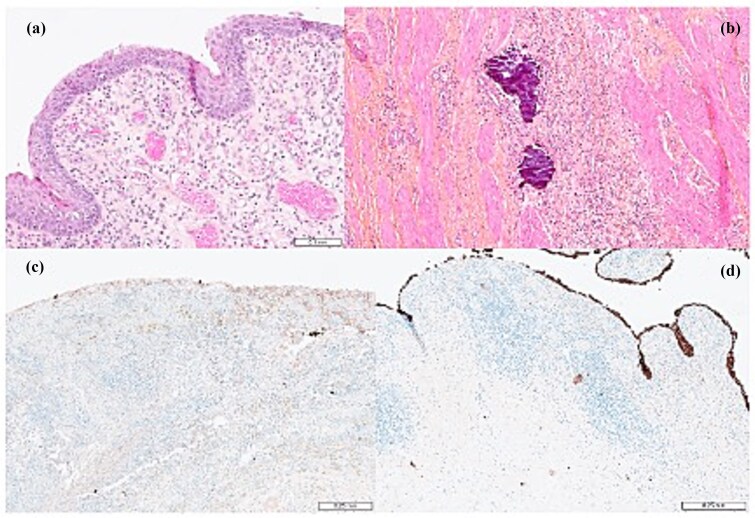
Histopathological examination of the RCP specimen showing no residual viable tumor cells, consistent with a pCR (ypT0). (a) Regenerative urothelial mucosa overlying granulation tissue, without residual viable tumor cells, consistent with treatment-related changes. (b) Inflammatory infiltrate with dystrophic calcifications within the former tumor bed, reflecting post-therapeutic changes after systemic treatment. (c and d) Immunohistochemical staining with pancytokeratin AE1/AE3 showing no residual carcinoma cells within the granulation tissue. Normal staining of the superficial urothelial cells is observed, confirming the absence of viable tumor cells.

Postoperative recovery was uneventful. Given the sustained response and the patient’s good performance status, the multidisciplinary team decided to continue EVP for a planned total duration of 1 year with radiological reassessment every 3 months. The first two follow-up imaging assessments at 3 and 6 months showed no evidence of disease recurrence.

Resumption of treatment was complicated by grade II cutaneous toxicity requiring a further reduction of EV to 0.75 mg/kg.

## Discussion

The management of mMIBC has historically relied primarily on systemic therapy because of the poor prognosis of metastatic disease and the substantial morbidity associated with RCP in this setting [5, 6]. During the chemotherapy era, surgery was therefore mainly restricted to palliative indications or highly selected situations. Nevertheless, several retrospective studies suggested a potential survival benefit of cytoreductive RCP in carefully selected patients receiving systemic chemotherapy. In a large observational study based on the National Cancer Database, Xu *et al.* [7] reported improved overall survival in patients undergoing cytoreductive RCP compared with conservative local management, particularly in patients with limited metastatic burden.

Patient selection remains a major challenge in this context. Metastatic burden appears to be an important prognostic factor, with improved outcomes reported in patients with a single metastatic site compared with those with multiple lesions [8]. In addition, emerging biomarkers such as circulating tumor DNA (ctDNA) may help refine risk stratification. Recent data from the IMvigor011 trial highlighted the strong prognostic value of ctDNA clearance after definitive local therapy in MIBC, suggesting that molecular assessment could complement imaging-based evaluation in identifying patients achieving minimal residual disease negativity [9, 10]. However, prospective validation of these approaches in the metastatic setting remains lacking.

The therapeutic landscape of metastatic urothelial carcinoma has recently changed with the introduction of antibody–drug conjugate–based combinations. EVP is now the standard first-line treatment following the results of the EV-302 trial, which demonstrated substantial improvements in response rates and overall survival compared with platinum-based chemotherapy [1, 2]. In the perioperative setting, the KEYNOTE-905/EV-303 trial also reported markedly improved pCR rates and event-free survival with EVP in cisplatin-ineligible MIBC [11]. These findings suggest that modern systemic therapies may achieve deeper and more durable responses, potentially opening the door to response-guided local treatment strategies.

Similar concepts have previously emerged in metastatic renal cell carcinoma, where the role of cytoreductive surgery has been redefined in the era of effective systemic therapy. The CARMENA and SURTIME trials demonstrated that deferred cytoreductive nephrectomy after initial systemic treatment may benefit carefully selected responders [12, 13]. This response-adapted strategy provides a conceptual framework for reconsidering the role of consolidative surgery in metastatic urothelial carcinoma.

In this context, the present case illustrates a rare but clinically meaningful scenario in which *de novo* mMIBC treated with first-line EVP achieved a mCR that was subsequently confirmed as a pCR after consolidative RCP. Although surgery is not currently recommended in mMIBC, this observation highlights the potential role of deferred local treatment in carefully selected patients achieving deep and durable responses to modern systemic therapies [14]. Prospective studies will be required to clarify patient selection, optimal timing, and the potential survival benefit of such an approach.

## Conclusion

This case illustrates a rare scenario of *de novo* metastatic MIBC achieving a complete pathological response after first-line EVP followed by consolidative cystoprostatectomy. Although surgery is not currently recommended in metastatic bladder cancer, the depth and durability of responses achieved with modern systemic therapies raise the question of response-guided local treatment in carefully selected patients. Prospective studies are required to clarify the role, timing, and patient selection for consolidative surgery in this setting.
